# Supplementary material for: Basics of faculty-to-faculty mentoring: A process to identify support and challenges
Source: PLoS One. 2023 Jun 13;18(6):e0287127. doi: 10.1371/journal.pone.0287127 (PMC10263330; doi:10.1371/journal.pone.0287127)
Supplement: Attachment — Submitted filename: Response to reviewers.docx [file pone.0287127.s002.docx]

**January 18, 2023**

Editor PLOS ONE,

Subject: Submission of Revised Manuscript **PONE-D-22-31892**

Dear Editor,

I would like to submit my revised vmanuscript titled as a research article “Basics of Faculty-to-Faculty Mentoring: A Process to Identify Support and Challenges”. Following are the changes made as suggested

Regards

Dr Tabassum Zehra

Assistant professor

Director Master of Health Professions Education

Aga Khan University

| **Editor’s Comment** | **Revision** |
| --- | --- |
| Please ensure that your manuscript meets PLOS ONE's style requirements | **Done and followed** |
| In the ethics statement in the Methods, you have specified that verbal consent was obtained. Please provide additional details regarding how this consent was documented and witnessed | **Revised, added details** |
| Upon re-submitting your revised manuscript, please upload your study’s minimal underlying data set | **Data Availability statement**  **Data is available with the authors. Data can be accessed as a repository** |
| Please amend either the title on the online submission form (via Edit Submission) or the title in the manuscript so that they are identical. | **Done** |
| **First Reviewer’s comment** |  |
| This study’s objectives are unclear and not mentioned anywhere in the manuscript and thus cannot be correlated with the title. It is unclear whether the objective is to report the process of the workshop or to share the perception of the involved participants about the mentoring program. | **Added** |
| The introduction needs major revision as it is not written in a systematic way e.g. there are two places in the first paragraph where there is a mention of a “dearth of literature” but the writing in between these two mentions has no link with them. It should be written in a crisp and organized way. | **Revised as suggested** |
| It is unclear from the manuscript that the intervention is done before the launch of the formal mentoring program in 2019. Not mentioning the timelines gave a sense of confusion in understanding the findings e.g. were the recognized challenges were difficulties that the faculty encountered or potential that they recognized before participating in the program? | **Revised as suggested** |
| The methodology section does not adequately describe the procedure. Even providing an example of a single scenario employed will allow readers to comprehend the process. The duration and timeline of the research process (workshop) should be mentioned when reporting it. It is important to explain how the scenario led to the conclusions and the method utilized to analyze the information from the workshop discussion. | **Scenarios added. Revision made** |
| In the result section, the key to the asterisk (*) is not mentioned in table 1. | **Removed** |
| **Second Reviewer’s comments** |  |
| Research question should be more focused and clear. | **Added** |
| There are many stakeholders, and needs-assessment cannot be identified with one workshop and with the inclusion of only one stakeholder, the faculty mentors. Other stakeholders, e.g., junior faculty members and institutional leadership should be approached as well to strengthen the findings. | **Added in the limitations and way forward** |
| If feasible, in-depth qualitative part can be included in methodology to give better interpretations. | **Added in way forward** |
| It is mentioned in the study that formal faculty mentorship program was initiated by the institution, and there is no standard faculty mentorship model to be followed. This point should be elaborated further, as, mostly, when an institution starts a formal mentorship program, it mentions mentorship model in detail with standard operating procedures (SOP). | **Identified the need for the model and revised** |
| Minor corrections are needed, e.g., full stop (.) is missing in line no. 4 of Abstract after "creating a risk of unexpected outcome", and no. of participants is twenty-two, while, in results section twenty is mentioned. | **Done** |
| Most of the findings of the research is already present in literature. Authors should highlight additional findings in the local context. | **Done** |
