# Supplementary material for: Basics of faculty-to-faculty mentoring: A process to identify support and challenges
Source: PLoS One. 2023 Jun 13;18(6):e0287127. doi: 10.1371/journal.pone.0287127 (PMC10263330; doi:10.1371/journal.pone.0287127)
Supplement: Attachment — Submitted filename: Response to the editor 28-03-2023.docx [file pone.0287127.s003.docx]

**March 28, 2023**

Editor PLOS ONE,

Subject: Submission of Revised Manuscript **PONE-D-22-31892**

Dear Editor,

I would like to submit my revised manuscript titled as a research article “Basics of Faculty-to-Faculty Mentoring: A Process to Identify Support and Challenges”. Following are the changes made as suggested

Regards

Dr Tabassum Zehra

Assistant professor

Director Master of Health Professions Education

Aga Khan University

| **Editor’s Comment** | **Revision** |
| --- | --- |
| Please ensure that your manuscript meets PLOS ONE's style requirements | **Further reviewed and revised the language** |
| In the ethics statement in the Methods, you have specified that verbal consent was obtained. Please provide additional details regarding how this consent was documented and witnessed | **Revised, further details added** |
| Upon re-submitting your revised manuscript, please upload your study’s minimal underlying data set | **Data Availability statement**  **Data is available with the authors. Data can be accessed as a repository** |
| Please amend either the title on the online submission form (via Edit Submission) or the title in the manuscript so that they are identical. | **Done** |
